# Cataract surgery: a public health crisis with your name on it

**DOI:** 10.1038/s41433-024-03502-6

**Published:** 2024-11-26

**Authors:** John D. Ellis, Obaid Kousha, Omkaar Sivanesan, Whitney Murray

**Affiliations:** 1 Department of Ophthalmology, Ninewells Hospital, NHS Tayside, James Arrott Dr, Dundee, DD2 1SG UK; 2 Infection & Global Health Division, School of Medicine Medical and Biological Sciences Building, North Haugh, St Andrews, KY16 9TF UK

**Keywords:** Lens diseases, Epidemiology

Cataract surgery, like air travel, and good sewerage, is amongst modernity’s greatest gifts. The Organisation for Economic Co-operation and Development reports that 20% of all healthcare in its 38 member Nations has no perceived benefit to its recipients [1]. In contrast a prospective study with over 23,500 person-years of follow up, showed that cataract surgery reduces the risk of dementia by 30% with benefit preserved up to ten years [2]. The return on investment is profound, measured as 4500% in direct and indirect medical costs, non-medical costs and contribution to national wealth/GDP [3]. Furthermore, health gains and economic viability have been consistently demonstrated for both first and second eyes [4, 5], and the reduction in falls risk [6], and preservation of independence hardly need stated. NICE failed to find any acuity threshold at which cataract surgery was not cost effective in a symptomatic patient [5]. Given that cataracts are only a reward for living long enough, they are really a *locus classicus* of a public health problem needing a public health solution.

Of the United Kingdom’s 7.64 million citizens currently on a waiting list [7], those with cataract experience widely divergent waits depending on wealth, geography and the presence or absence of co-pathology [8]. Post austerity and pandemic, NHS patient services have struggled. The UK has the fastest rise in healthcare expenditure from out-of-pocket or voluntary insurance sources in the G7 countries [9].

The Independent Sector Treatment Centres (ISTCs) have ‘helped’ at considerable expense, doing over half the cataract surgeries, impacting training, less lucrative aspects of ophthalmology, and leaving behind those with the most complex needs. 58% of eye units say these providers are having a negative impact on their service [8].

Are the ISTCs the best solution for the taxpayer? Possibly there are untapped efficiency gains to be had in recognition of the eye’s paired organ status. Immediate Sequential Bilateral Cataract Surgery (ISBCS) is difficult to argue against but easy to emote against. In 2021–22 the National Ophthalmic Database recorded 1463 cases performed in England and Wales, contributed by 267 surgeons [10]. Reservations relate to bilateral complications which would be catastrophic to the patient. Bilateral simultaneous endophthalmitis is low on the list of probability [11]. When total instrument separation, re-scrubbing and separate batch numbers of supplies between eyes are adhered to the risk is one in 28.5 million [11, 12]. To contextualise this, the *average* patient (age 76), driven by an *average* spouse an *average* round-trip for the second eye is over 50 times more likely to be killed or seriously injured in a road traffic accident [13].

**Fig. 1:**
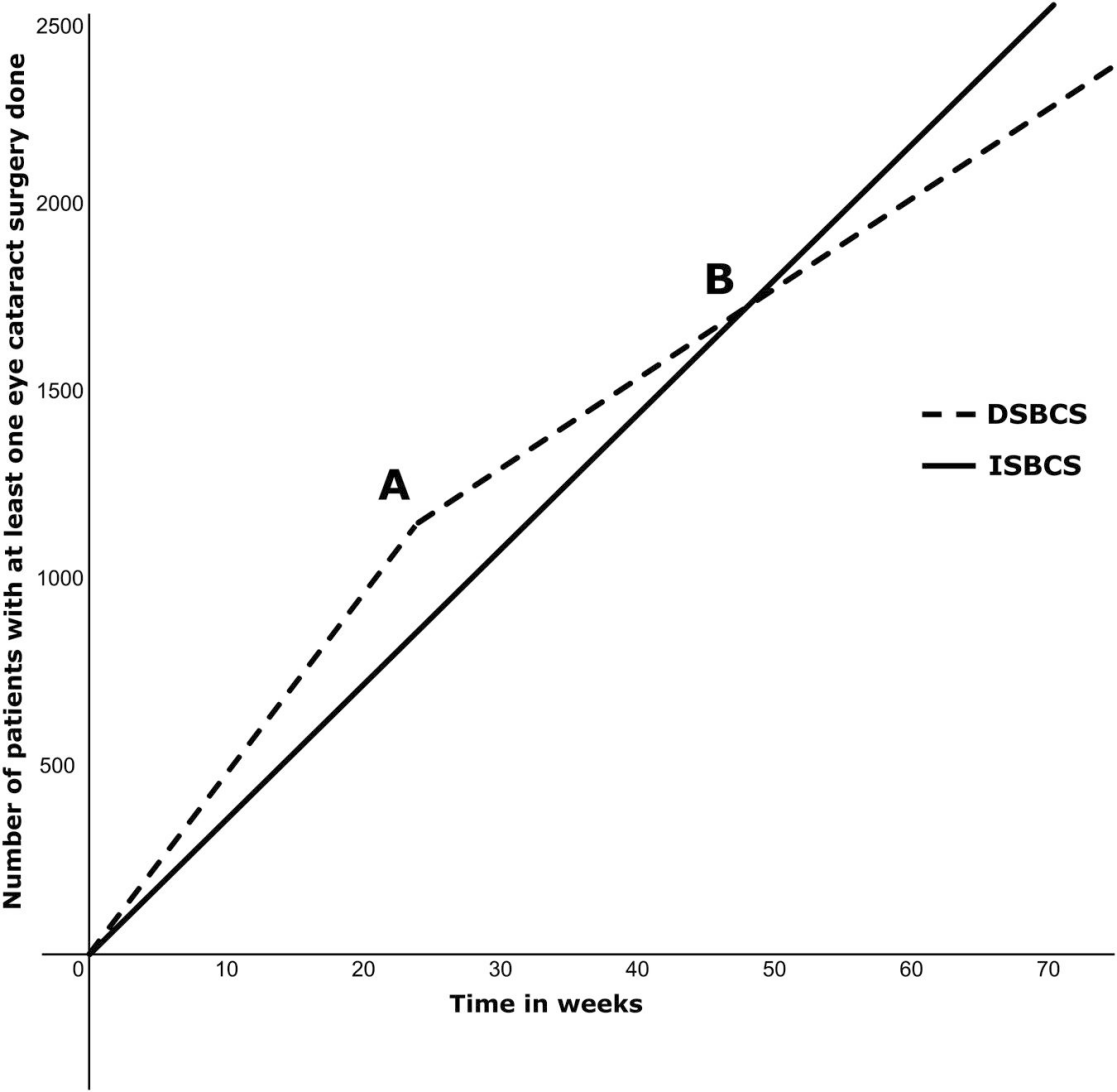
Number of patients who have had at least one eye surgery over time; modelled with 24 week referral to clinic time in treatment naive population with bilateral cataracts on first eye listing. “Initially more patients have at least one eye done in DSBCS group until point (**A**) when second eyes start to be referred to hospital; binocularly treated patients slows overall rate”. “**B** Point at which theatre efficiency gains with ISBCS means that number of patients with at least one eye done overtakes DSBCS with added benefit of being binocularly treated at the same time”.

Offering some patients the option of both eyes being ‘helped’ when some haven’t been ‘helped’ even with their first, raises an ethical dilemma. To steel-man this argument, a population in which all had at least one eye done would manage at home whilst waiting their second, whilst ISBCS adoption means half that same population are sighted, the rest remaining benighted. This argument fails philosophically and practically. The former because no hospital has a policy that no one gets a second eye (delayed sequential bilateral cataract surgery (DSBCS)) till all in the queue have had their first done. It fails practically because the waiting list runs faster with ISBCS. Efficiency gains in theatre mean that in whole day/two sessions, we consider 15 patients; 30 eyes (ISBCS) and 24 patients; 24 eyes (DSBCS) to be time equivalent. These numbers are below absolute time-limits to comfortably allow enough time for training. Mathematical modelling [Fig. 1] shows that the optometrist to clinic listing time, and the ability to do more eyes on lists leads to a crossover-point in time from a department starting ISBCS, after which the numbers done overtake a DSBCS only department. For us with optometrist to clinic time 24 weeks, this inflection occurred around 48 weeks. We are now in week 130 and the advantages are all in favour of ISBCS at the population level.

The apprehension regarding refractive surprise and lost benefit of adjusting the target for the second eye being lost with ISBCS has been answered. A robust Dutch study found no difference in second eye refractive outcomes of ISBCS and DSBCS [14].

One final concern is the dreaded complication of intractable macular oedema. Given that transient oedema is common but usually clears by week 4-6, early review would be too soon to determine which patients will suffer. One UK study found this to persist at one year in 0.02% of patients [5]. This is notable because a study of ISBCS from the US reported 44 525 immediate surgeries. But they also reported a further 897 469 cases of DSBCS where the second eye was operated within 14 days of the first [15]. Whilst the latter would all but eliminate the risk of BSPOE it offers none of the time and motion economies of ISBCS. With respect to the risk of bilateral macular oedema it amounts to *forme fruste* ISBCS but is philosophically incoherent, offering the full advantages of neither immediate nor delayed surgery.

In closing it must be reported that tragedy has struck, with a single list of ISBCS cases in a community-based clinic in Denmark where contamination with *S epidermidis* caused 6 cases of endophthalmitis in 3 patients. One eye was lost, and although the remaining eyes all achieved very good vision [16], this is extremely sobering. Returning by way of metaphor to those gifts of modernity that we take for granted, the response to an air disaster is grief and adaptation, with improved safety, enforced if necessary. Ground planes whose doors fall off, but don’t ground all planes forever. In the case of flying, it is not possible to divide your investments. You are all in, and what you need is knowledge of what constitutes fair and reasonable risk for a desirable outcome. We need to do the same for cataract surgery in the UK. Let’s enforce safety and quality and do our best for the UK. And in the end for ourselves.
